# SE-SNN: Squeeze-and-Excitation-Enhanced Spiking Neural Networks with Learnable Neuron Dynamics for Event-Based Vision

**DOI:** 10.3390/biomimetics11050359

**Published:** 2026-05-21

**Authors:** Chuang Liu, Yang Chen

**Affiliations:** School of Intelligent Science and Information Engineering, Shenyang University, Shenyang 110044, China

**Keywords:** spiking neural networks, neuromorphic computing, squeeze-and-excitation, learnable neuron dynamics, event-based vision, CIFAR10-DVS

## Abstract

Spiking neural networks (SNNs) have emerged as a promising paradigm for energy-efficient neuromorphic computing, particularly when processing asynchronous event streams from dynamic vision sensors (DVSs). However, SNNs often suffer from limited representational capacity and suboptimal feature recalibration compared to their artificial counterparts. To address these challenges, we propose SE-SNN, a novel architecture that integrates Squeeze-and-Excitation (SE) blocks into deep residual SNNs, enabling channel-wise attention without spike generation. Furthermore, we introduce a Robust Parametric Leaky Integrate-and-Fire (RobustPLIF) neuron model with learnable membrane time constant (τ) and firing threshold ($v_{\mathrm{th}}$), allowing adaptive temporal dynamics in each layer. Our model is trained on the CIFAR10-DVS dataset.The experimental results demonstrate that SE-SNN achieves an accuracy of 78.8 % on CIFAR10-DVS with 16 time steps, outperforming baseline SNNs while maintaining biological plausibility and hardware efficiency. Ablation studies confirm the individual contributions of the SE blocks and learnable neuron parameters to the performance gains.

## 1. Introduction

Spiking neural networks (SNNs) have garnered extensive attention in recent years owing to their superior biological interpretability, low power consumption, and low latency. As a distinctive feature, SNNs employ discrete spike events for information transmission and exhibit sparse activation properties [1]. These characteristics not only enable ultra-low power consumption and low latency but also endow SNNs with a unique capability to capture key features in dynamic time-series data, thereby demonstrating enormous application potential [2]. Specifically, SNNs possess prominent event-driven sparsity: their neurons strictly adhere to a spike-triggered mechanism, where spike signals are generated only when the accumulated membrane potential exceeds the firing threshold. This biologically realistic “fire–rest” dynamic trait allows the neural network to maintain highly sparse activation in both spatial and temporal dimensions (i.e., spatiotemporal sparsity), and measurements have shown that it can reduce synaptic operation energy consumption by up to 60–80% [3]. Consequently, SNNs outperform traditional artificial neural networks (ANNs) in processing dynamic and continuous signal data, particularly displaying obvious advantages in tasks requiring efficient temporal feature processing, such as action recognition and speech processing [4,5]. With the continuous advancement of computing power, SNNs have been widely applied in various fields including image processing and signal recognition, further highlighting their great potential in low-power and efficient computing [6,7].

As the third generation of neural networks, SNNs differ significantly from traditional ANNs in terms of their constituent units, input–output methods, and operating mechanisms [8]. SNNs use spike signals as information carriers, and their neurons transmit and process information through discrete spike events, exhibiting strong temporal dynamic characteristics that are closer to the operating mechanisms of biological nervous systems in multiple aspects [9]. Firstly, SNNs adopt spike signals as the basic unit for information transmission, encoding information through the timing and frequency of spike emissions. This discrete, time-dependent information transmission method enables them to process spatiotemporal dynamic data more efficiently [10]. Secondly, neuronal activity in SNNs is sparse and event-driven, with spikes emitted only when receiving input signals of sufficient intensity, which confers significant low-power consumption advantages. Additionally, the learning algorithms of SNNs are based on biological principles, enabling more natural neural plasticity. These characteristics enable SNNs to exhibit great potential in simulating brain neural signal processing, handling complex spatiotemporal tasks, and achieving low-power computing, thus being widely recognized as an important direction in neural network development and representing the core features of the third generation of neural networks [11].

Currently, the learning methods of SNNs are mainly categorized into two types: the ANN-to-SNN conversion method and direct training [12]. Traditional ANNs inherently involve redundant computations, which inevitably increase computational costs during data processing. Both the conversion of ANNs to SNNs and the direct training of SNNs based on backpropagation supervised learning rules require substantial labeled data for model training, accompanied by high computational overhead. These methods still exhibit a considerable gap compared with the event-driven, efficient information processing mechanisms of biological neural systems. Despite recent advancements in conversion-based approaches and direct training methods, SNNs still lag behind ANNs in accuracy when tackling complex visual tasks. Two key limitations account for this performance gap: (1) the fixed dynamics of neurons fail to adapt to layer-specific feature statistics; and (2) the lack of explicit mechanisms for modeling inter-channel dependencies, which is critical for discriminative feature learning [13].

Moreover, SNNs still face significant challenges in training algorithm performance, parameter optimization, and network architecture design, which severely restrict their performance improvement and use in practical applications [14]. Specifically, the existing challenges of SNNs include the lack of effective training algorithms, the need for refined parameter optimization, and the requirement for adaptive adjustments to network architectures. Research on training algorithms [15] primarily addresses the issue that gradient descent algorithms cannot be directly applied to SNNs due to the non-differentiable nature of spike firing functions. Research on parameter optimization [16] aims to further enhance the accuracy and reduce the latency of SNNs. In terms of network architecture adjustments [17], the asynchronous information processing driven by spike events differs significantly from the synchronous continuous-value processing in ANNs. Therefore, when leveraging ANN architectures to construct SNNs, corresponding modifications to the network architecture are often indispensable. Typical adjustments include improvements to residual connections, pooling layers, and batch normalization methods. These adjustments adapt to the characteristics of spike signals by redesigning the input and output forms of each layer, thereby enabling the effective application of such architectures in SNNs [18]. Nevertheless, existing SNN architectures still have numerous shortcomings. Some architectures merely treat spiking neurons as a special type of activation function, ignoring the temporal correlation between spikes and thus failing to fully utilize the spatiotemporal characteristics of SNNs. Others neglect the binary nature of spike sequences during information transmission, leading to inaccurate inter-layer data propagation and even information loss [19].

Event-based cameras, such as dynamic vision sensors (DVSs), capture visual information as asynchronous streams of sparse “events” triggered by pixel-level intensity changes. This paradigm offers the advantages of higher temporal resolution, lower latency, and greater energy efficiency compared to conventional frame-based cameras [20]. Inspired by biological neural systems, SNNs naturally align with such asynchronous data due to their event-driven computation and temporal coding capabilities. We implemented our model using the SpikingJelly framework [21] and evaluated it on the challenging CIFAR10-DVS dataset [22]. Our training protocol incorporates Mixup data augmentation and exponential moving average (EMA), and robust learning rate scheduling to stabilize the optimization process. Our architecture integrates a spiking-adapted Squeeze-and-Excitation (SE) module, inspired by the seminal work of Hu et al. [23]. In CNNs, the SE block enhances representational capacity by explicitly modeling inter-channel dependencies: it squeezes spatial information through global average pooling and excites informative channels via a learnable gating mechanism. While highly effective in ANNs, direct application to SNNs is infeasible due to the discrete, binary nature of spike activations. To address this issue, we compute the squeeze statistic using the time-averaged spike count per channel and implement excitation as a multiplicative scaling factor applied directly to the membrane potential prior to spike generation. This design retains the core principle of channel-wise attention while fully respecting the event-driven characteristics of spiking neural networks.The proposed SE-SNN achieves competitive performance with minimal computational overhead, demonstrating the effectiveness of attention mechanisms and adaptive neuron models in SNNs.

To bridge the aforementioned gaps, we propose three synergistic innovations:

- A learnable robust PLIF neuron (RobustPLIF) with trainable τ and $V_{\mathrm{th}}$, enabling automatic adjustment of temporal integration and spiking behavior.
- Integration of Squeeze-and-Excitation (SE) blocks into SNN residual blocks, where the SE module operates on membrane potentials to generate channel-wise attention weights via standard differentiable operations.
- The experimental results demonstrate that SE-SNN achieves a state-of-the-art accuracy of 78.8% on CIFAR10-DVS.

The remainder of this paper is organized as follows: Section 2 presents a brief review on background of learning and encoding strategies, architectural innovations, and adaptive neuron models for SNNs. In Section 3, the details of the proposed algorithm are elaborated. The comprehensive study and experimental results are discussed in Section 4, and finally, Section 5 provides the concluding remarks of the study.

## 2. Related Work

SNNs have garnered increasing attention as a promising paradigm for energy-efficient neuromorphic computing, owing to their event-driven, sparse, and asynchronous processing characteristics. While early SNNs were primarily constrained by shallow architectures and biologically inspired but limited learning rules like spike-timing-dependent plasticity (STDP) [24], recent advances in surrogate gradient-based training [25] and ANN-to-SNN conversion techniques [26] have enabled the construction of deep SNNs that rival ANNs in accuracy on standard vision benchmarks such as CIFAR-10/100 and ImageNet [27]. However, directly transplanting ANN architectural components—such as residual connections or attention modules—into SNNs often fails due to the fundamental differences in signal representation: ANNs operate on continuous real-valued activations, whereas SNNs communicate via discrete, binary spikes over time. This section reviews key developments in three inter-related dimensions that underpin modern deep SNN design: (1) learning and encoding strategies, (2) architectural innovations for depth and feature recalibration, and (3) adaptive neuron models.

### 2.1. Learning Paradigms and Temporal Encoding

**Figure 1:**
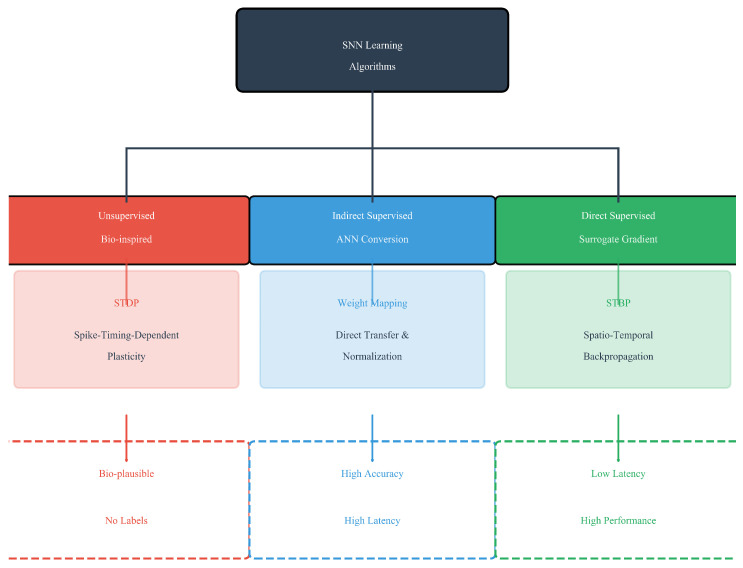
Taxonomy of SNN learning algorithms. The three main branches are (1) biologically plausible unsupervised learning (e.g., STDP); (2) indirect supervised learning via ANN-to-SNN conversion; and (3) direct supervised learning using surrogate gradient methods.

SNNs require mechanisms to convert real-valued inputs into spike trains, a process known as neural encoding [28]. While various schemes exist (e.g., rate coding, temporal coding), most deep SNNs for static vision tasks adopt simple rate coding, where pixel intensities are proportional to firing probabilities over a fixed simulation window [29]. The translation of analog signals into discrete spike trains, known as neural encoding, constitutes a fundamental operation in SNNs. Unlike conventional ANNs that process continuous-valued activations, SNNs rely on sparse, binary spike events, necessitating explicit encoding mechanisms to interface with real-world sensory data [30]. The choice of encoding strategy profoundly impacts the trade-off between computational efficiency, temporal precision, and biological plausibility. Training deep SNNs remains challenging due to the non-differentiability of the spiking function. The development of effective learning algorithms represents the central challenge in advancing SNNs. The non-differentiable nature of the spike generation function (typically a Heaviside step function) precludes the direct application of standard backpropagation, necessitating specialized training methodologies [25]. Current approaches can be taxonomically divided into three principal categories: biologically inspired unsupervised learning, indirect supervised learning via ANN conversion, and direct supervised learning using surrogate gradients (as illustrated in Figure 1).

Table 1 provides a comparative overview of learning algorithms.

- Unsupervised local learning, exemplified by STDP [24], enables self-organization but struggles with scalability and task-specific optimization. Unsupervised learning algorithms in SNNs draw inspiration from biological synaptic plasticity mechanisms. STDP adjusts synaptic weights based on the precise temporal correlation between pre- and post-synaptic spikes: if a presynaptic spike precedes a postsynaptic spike, the synapse is potentiated; otherwise, it is depressed. This local learning rule requires no labeled data and can be implemented in an event-driven, online fashion, making it highly attractive for neuromorphic hardware deployment. Variants of STDP, including reward-modulated STDP (R-STDP) and triplet-STDP, have been developed to stabilize learning and incorporate global reward signals. However, purely STDP-based approaches are generally limited to simple feature extraction and clustering tasks, struggling with complex pattern recognition due to the absence of global error signals. Consequently, unsupervised STDP is often employed for pre-training or feature learning in hybrid training pipelines.
- Indirect supervised learning via ANN-to-SNN conversion maps pre-trained ANNs to SNNs by replacing ReLU units with integrate-and-fire (IF) or leaky integrate-and-fire (LIF) neurons. ANN-to-SNN conversion offers an alternative pathway to obtain high-performance SNNs without directly confronting the non-differentiability problem. This approach involves training an equivalent ANN using conventional backpropagation, followed by mapping the trained weights and architecture to an SNN with rate-based coding. The conversion process typically requires replacing ReLU activations with IF neurons and adjusting firing thresholds to match ANN activation distributions. Conversion methods have achieved near-lossless accuracy on ImageNet and other challenging datasets, often utilizing hundreds to thousands of time steps to approximate real-valued activations with spike rates. Recent advances include threshold-balancing techniques, weight-normalization strategies, and layer-wise calibration methods to minimize conversion error. However, the primary limitation remains high inference latency: the converted SNNs require extensive temporal windows to accumulate sufficient spike counts for accurate prediction, diminishing the energy efficiency and rapid inference benefits inherent to SNNs [31].
- Direct supervised learning using surrogate gradients [25] has become the dominant method for low-latency SNNs. By approximating the derivative of the spike-generation function during backpropagation, it enables end-to-end training of deep SNNs within tens of timesteps. This breakthrough has facilitated the adoption of complex architectures such as ResNets [27] and Vision Transformers  [33]. Direct training of SNNs via gradient descent has emerged as the dominant paradigm for achieving high performance with low latency [25]. The key innovation is the surrogate gradient (SG) method, which circumvents the non-differentiability of spike generation by substituting the derivative of the Heaviside function with a smooth approximation during the backward pass.

### 2.2. Architectural Innovations for Deep SNNs

The architectural design of SNNs has evolved significantly, transitioning from shallow biologically inspired models to deep, high-performance structures capable of competing with conventional ANNs on complex tasks. Modern SNN architectures can be broadly categorized into feedforward networks, recurrent architectures, and hybrid structures incorporating attention mechanisms.

#### 2.2.1. Feedforward and Convolutional Architectures

Feedforward SNNs, particularly spiking convolutional neural networks, represent the most prevalent architecture for static image classification tasks. These networks typically employ LIF neurons within convolutional layers to extract hierarchical spatial features. However, the direct application of traditional residual connections to SNNs often results in network degradation as depth increases, primarily due to the non-differentiable nature of spike generation [34].

To address these limitations, Fang et al. [34] proposed SEW-ResNet (Spike-Element-Wise ResNet), which modifies residual blocks to accommodate binary spike activations, enabling successful training of deep SNNs with over 50 layers. Concurrently, Hu et al. [35] introduced alternative residual block designs specifically optimized for spiking neurons. Zheng et al. [36] further extended network depth by proposing threshold-dependent batch normalization, which calibrates neuronal thresholds adaptively during training. These architectural innovations have substantially narrowed the performance gap between deep SNNs and ANNs on benchmarks such as CIFAR-10 and ImageNet.

#### 2.2.2. Recurrent Architectures and Reservoir Computing

Recurrent SNNs incorporate feedback connections that enable the maintenance of internal states and memory of past inputs, making them inherently suitable for temporal sequence processing [37]. A prominent example is the Liquid State Machine (LSM), a reservoir computing paradigm proposed by Maass et al. [38]. The LSM consists of a randomly connected recurrent network of spiking neurons (the “liquid”) that projects input signals into a high-dimensional dynamical space, followed by a trainable readout layer.

The LSM exhibits the fading memory property, allowing it to retain information about past inputs beyond the short-term integration time constants of individual neurons [38]. Recent work has explored hybrid architectures combining LSM with unsupervised learning mechanisms such as spiking self-organizing maps (SOMs) for visual clustering, achieving competitive performance on MNIST and speech recognition tasks without labeled data [39]. Additionally, echo state networks (ESNs) with spiking neurons have been investigated, where fixed random weights satisfying the echo state property ensure that network dynamics are driven predominantly by input signals.

Despite their biological plausibility and computational capabilities, recurrent SNNs present significant challenges in training due to complex dynamics and the difficulty of credit assignment through time.

#### 2.2.3. Transformer and Attention-Based Architectures

The success of attention mechanisms in ANNs has motivated their adaptation to SNNs. Spiking Vision Transformers (SVTs) and spatio-temporal self-attention mechanisms have been developed to capture long-range dependencies in visual data [40]. Recently, Zhou et al. [41] proposed Spikingformer, which integrates bio-plausible spiking dynamics with transformer architectures, demonstrating that attention mechanisms can be effectively implemented with sparse spike-based communications.

Hybrid architectures combining convolutional and attention layers have also emerged. For instance, the Efficient Spatio-Temporal Spiking Transformer (ESTSformer) optimizes the trade-off between computational cost and representational capacity by leveraging factorized attention mechanisms [42]. These architectural innovations have expanded the applicability of SNNs to tasks requiring global context understanding, such as video analysis and multi-modal fusion.

### 2.3. Adaptive and Learnable Neuron Models

The choice of neuron model fundamentally shapes an SNN’s computational capabilities. Fixed-parameter LIF neurons, while biologically plausible, lack flexibility for diverse layer-wise feature statistics. The Parametric LIF model is an important evolution of the LIF model in SNNs. Its core breakthrough lies in transforming the originally fixed membrane time constant into a trainable parameter, thereby endowing neurons with the capability of adaptive temporal dynamics. Recent works advocate for learnable neuron dynamics. Wu et al. [43] introduced the Parametric LIF neuron, where the membrane time constant $\tau_{m}$ is a learnable parameter for each layer, enabling adaptive temporal integration.

#### 2.3.1. Traditional LIF and Its Limitations

The LIF model is one of the most widely adopted spiking neuron models in SNNs due to its computational simplicity and biological interpretability. This rigidity limits the neuron’s ability to adapt its temporal integration window to different input statistics or task requirements, often resulting in suboptimal performance when processing complex spatiotemporal data.

#### 2.3.2. Emergence of Parametric LIF

In traditional LIF models, the membrane time constant τ is a predefined hyperparameter that determines the decay rate of the membrane potential but remains non-updatable during training. The PLIF model—systematically proposed by Fang et al. at ICCV 2021—introduces the following key modifications [27]:

- Learnable time constant: A trainable variable is introduced and mapped to the decay rate k(a) or time constant τ via sigmoid or exponential transformations. For instance, τ=1+exp(−a), where *a* is optimized alongside network weights using backpropagation algorithms such as Spike-Timing-Dependent Backpropagation (STBP).
- Biological plausibility: This design mimics the neuronal heterogeneity observed in biological brains, allowing different layers or neurons to learn distinct “memory lengths” or response frequencies according to task requirements.

To address this limitation, recent studies have focused on parameterizing the neuronal dynamics. This design allows τ to be optimized jointly with synaptic weights using gradient-based methods. Compared to fixed-τ LIF, PLIF not only improves accuracy on both static datasets (e.g., CIFAR-10) and neuromorphic datasets (e.g., DVS128 Gesture) but also reduces the number of required simulation timesteps, thereby lowering the inference latency and energy consumption.

Building on this, our Robust PLIF model further introduces a learnable firing threshold $v_{\mathrm{th}}$, jointly optimized with $\tau_{m}$. This dual-parameter adaptation allows each layer to independently regulate both its temporal smoothing and spiking sensitivity—a capability shown to improve robustness to input noise and distribution shifts.

## 3. Methodology

**Figure 2:**
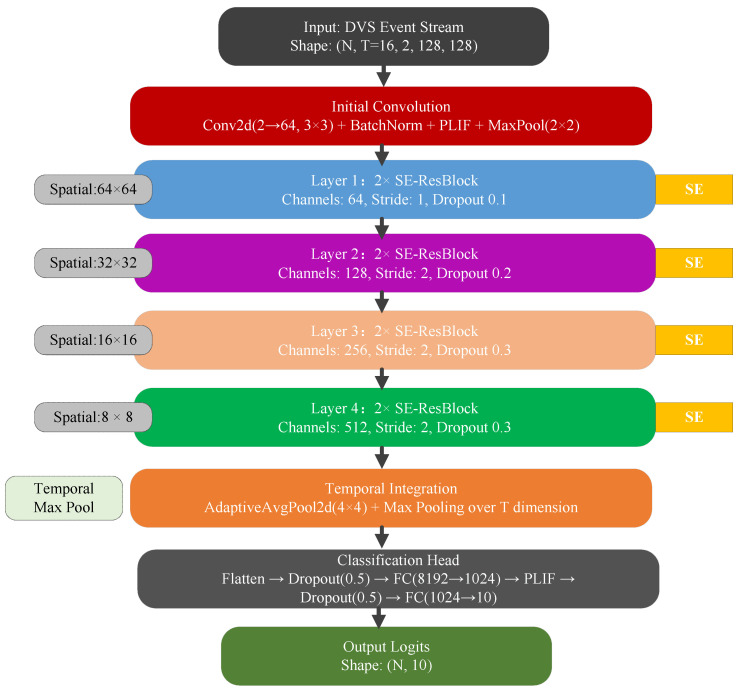
Overall Architecture of SE-SNN for DVS-CIFAR10 classification. The SE module operates on membrane potentials.

In this section, we present the detailed architecture of our proposed SE-SNN (Squeeze-and-Excitation Spiking Neural Network), a deep residual SNN enhanced with channel attention mechanisms for robust event-based object recognition. We first introduce the robust neuron model, followed by the SE-ResNet architecture, temporal integration strategy, and comprehensive training pipeline. The overall architecture is shown in Figure 2.

The detailed architectural configuration of the proposed SE-SNN is summarized in Table 2.

### 3.1. Robust PLIF Neuron Model

Unlike conventional LIF neurons with fixed hyperparameters, we propose a Robust Parametric Leaky Integrate-and-Fire (RobustPLIF) neuron, where both the membrane time constant τ and firing threshold $v_{\mathrm{th}}$ are learnable parameters optimized during training.

Membrane dynamics: The subthreshold dynamics of our RobustPLIF neuron is given in Equation (1).

(1) $$\tau \frac{dv(t)}{dt}=-\left(v\left(t\right)-v_{\mathrm{rest}}\right)+I\left(t\right),$$

where $v_{\mathrm{rest}}=0$ is the resting potential, I(t) represents the input current, and $\tau \in R^{+}$ is the learnable time constant controlling the leakage rate. The resting potential is fixed at $v_{\mathrm{rest}}=0$, following standard convention in deep SNNs [27]. This choice simplifies the dynamics without loss of generality, as any non-zero resting potential can be equivalently represented by shifting the firing threshold.

Firing mechanism: When the membrane potential exceeds the learnable threshold $v_{\mathrm{th}}$, the neuron emits a spike. The expression is shown in Equation (2).

(2) $$s\left(t\right)=\Theta \left(v\left(t\right)-v_{\mathrm{th}}\right)=\left\{\begin{array}{cc} 1, & \mathrm{if}\;v\left(t\right)\geq v_{\mathrm{th}},\\ 0, & \mathrm{otherwise}, \end{array}\right.$$

where Θ( · ) is the Heaviside step function. After firing, the membrane potential is reset to $v_{\mathrm{rest}}$.

Surrogate gradient: To enable backpropagation through the non-differentiable spike function, we employ the *ArcTangent* surrogate gradient. The formula is presented in Equation (3).

(3) $$\frac{\partial s}{\partial v}\approx \frac{\alpha }{2\left[1+{\left(\frac{\pi }{2}\alpha \left(v-v_{\mathrm{th}}\right)\right)}^{2}\right]},$$

where α=2.0 is a constant controlling the steepness of the surrogate gradient. We adopt detach_reset=True to prevent gradient interference from the reset operation while preserving temporal dependencies during backpropagation.

Parameter constraints and their justification: To ensure numerical stability, meaningful spiking activity, and biologically plausible dynamics, the hard constraints on the learnable parameters are given in Equation (4).

(4) $$\tau \in \left[1.0,20.0\right],\;v_{\mathrm{th}}\in \left[0.2,0.8\right].$$

The hard constraints imposed on the learnable parameters are detailed below.

- For τ: In discrete-time simulation (with Δt=1), the decay factor is $\alpha_{\mathrm{decay}}=\exp\left(-1/\tau \right)$. If τ<1.0, then this causes rapid potential decay that eliminates temporal memory—defeating a key advantage of SNNs. Conversely, if τ>20.0, then this leads to near-zero leakage and unbounded potential accumulation, which induces numerical overflow and training instability [25]. The interval [1.0,20.0] ensures $\alpha_{\mathrm{decay}}\in \left[0.37,0.95\right]$, a range widely adopted in stable SNN designs [27].
- For $v_{\mathrm{th}}$: Our input event frames are normalized to [0,1], and intermediate features typically lie within [−1,2] during early training. A threshold below 0.2 would cause excessive firing even for weak inputs, saturating the network and degrading representational capacity. A threshold above 0.8 may suppress spiking entirely under typical activation magnitudes, resulting in dead neurons. The chosen range [0.2,0.8] aligns with the dynamic range of normalized features and matches empirical optima observed in deep SNNs trained on event-based vision tasks.

### 3.2. SE-ResNet Architecture

Our network follows a residual architecture with channel-wise attention modules specifically designed for spiking neural networks. The overall structure comprises an initial convolutional stem, four stages of SE-Residual blocks with progressive channel expansion, and a classification head with temporal aggregation. The forward-propagation implementation of SE-SNN is detailed in Algorithm 1.
**Algorithm 1** SE-SNN Forward Propagation**Require:** 
Event stream $X\in R^{N\times T\times 2\times H\times W}$, Network parameters θ, Time steps *T***Ensure:** 
Logits $Y\in R^{N\times N_{cls}}$  1:Initialize empty list O←[]  2:**for **t=1** to ***T ***do **  3:    $x_{t}\leftarrow X\left[:,t,:,:,:\right]$                   ▹ Extract *t*-th frame  4:    $h\leftarrow \mathrm{InitConv}(x_{t})$         ▹ Conv(2→64, 3 × 3) + BN + PLIF + MaxPool  5:    h←Layer1(h)                 ▹ SE-ResBlock ×2, 64 channels  6:    h←Layer2(h)            ▹ SE-ResBlock ×2, 128 channels, stride 2  7:    h←Layer3(h)            ▹ SE-ResBlock ×2, 256 channels, stride 2  8:    h←Layer4(h)            ▹ SE-ResBlock ×2, 512 channels, stride 2  9:    h←AdaptiveAvgPool2d(h,(4,4))10:    Append *h* to O11:**end for**12:H←stack(O,dim=1)                 ▹ Shape: [N,T,512,4,4]13:F←max(H,dim=1).values            ▹ Temporal max pooling14:Y←Classifier(F) ▹ Flatten + FC(8192→1024) + PLIF + Dropout + FC(1024→10)15:**return** *Y*

#### 3.2.1. Squeeze-And-Excitation for SNNs

Traditional SE blocks operate on activation maps in ANNs. In SNNs, we adapt this mechanism to operate on membrane potentials rather than binary spikes, preserving continuous information for effective channel recalibration.

Given an intermediate membrane potential tensor $X\in R^{N\times C\times H\times W}$ at a specific time step, the input is structured as a 5D tensor of shape (N,C,T,H,W), where *N* denotes the batch size, *C* the number of channels (e.g., 2 for ON/OFF polarities in DVS data), *T* the number of time steps, and H×W the spatial height and width. The SE module performs as follows.

Squeeze operation: Global average pooling aggregates spatial information into a channel descriptor $z\in R^{N\times C}$. The calculation procedure of the squeeze operation is illustrated in Equation (5).

(5) $$z_{c}=\frac{1}{H\times W}\sum_{i=1}^{H}\sum_{j=1}^{W}X_{c,i,j},\;c=1,\ldots ,C.$$

Excitation operation: A bottleneck architecture learns channel-wise dependencies. The excitation operation is shown in Equation (6).

(6) $$s=\sigma \left(W_{2}\;\cdot \;\mathrm{ReLU}\left(W_{1}\;\cdot \;z\right)\right),$$

where $W_{1}\in R^{\frac{C}{r}\times C}$ and $W_{2}\in R^{C\times \frac{C}{r}}$ are learnable weights with reduction ratio r=16, and σ(·) denotes the sigmoid function.

Scaling operation: The final output is obtained by channel-wise multiplication, and its calculation procedure is given in Equation (7).

(7) $${\tilde{X}}_{c,i,j}=s_{c}\;\cdot \;X_{c,i,j}.$$

#### 3.2.2. SE-Residual Block

Each residual block consists of two convolutional layers with SE attention and skip connections. The residual block is calculated as follows:

(8) $$\begin{array}{cc} h_{1} & =\mathrm{PLIF}(\mathrm{BN}\left({\mathrm{Conv}}_{3\times 3}\left(x\right)\right)), \end{array}$$

(9) $$\begin{array}{cc} h_{2} & =\mathrm{SE}(\mathrm{BN}\left({\mathrm{Conv}}_{3\times 3}\left(\mathrm{Dropout}\left(h_{1}\right)\right)\right)), \end{array}$$

(10) $$\begin{array}{cc} y & =\mathrm{PLIF}(h_{2}+F_{shortcut}\left(x\right)), \end{array}$$

where $F_{shortcut}$ is an identity mapping or 1×1 convolution with batch normalization when the spatial dimensions or channel numbers change.

The forward-pass implementation of the SE-Residual block is detailed in Algorithm 2.
**Algorithm 2** SE-Residual Block Forward Pass**Require:** 
Input membrane potential *x*, In channels $C_{in}$, Out channels $C_{out}$, Stride *s*, Use SE flag $I_{SE}$, Dropout rate *p***Ensure:** 
Output membrane potential *y*  1:identity←x  2:$out\leftarrow \mathrm{Conv}2d(x,C_{out},\mathrm{kernel}=3,\mathrm{stride}=s,\mathrm{padding}=1)$  3:out←BatchNorm2d(out)  4:out←PLIF(out)  5:**if **p>0** then**  6:    out←Dropout2d(out,p)  7:**end if**  8:$out\leftarrow \mathrm{Conv}2d(out,C_{out},\mathrm{kernel}=3,\mathrm{stride}=1,\mathrm{padding}=1)$  9:out←BatchNorm2d(out)10:**if **$I_{SE}=\mathrm{True}$** then**11:    z←AdaptiveAvgPool2d(out,(1,1))          ▹ Squeeze: global spatial avg12:    z←Flatten(z)                          ▹ Shape: $\left[N,C_{out}\right]$13:    $w\leftarrow \mathrm{Linear}(z,C_{out}//16)$                       ▹ Reduction14:    w←ReLU(w)15:    $w\leftarrow \mathrm{Linear}(w,C_{out})$                         ▹ Expansion16:    w←Sigmoid(w)                    ▹ Excitation: channel weights17:    $out\leftarrow out\times w.\mathrm{view}(N,C_{out},1,1)$                       ▹ Scale18:**end if**19:**if **s≠1** or **$C_{in}\neq C_{out}$** then**20:    $identity\leftarrow \mathrm{Conv}2d(x,C_{out},\mathrm{kernel}=1,\mathrm{stride}=s)$21:    identity←BatchNorm2d(identity)22:**end if**23:out←out+identity                     ▹ Residual connection24:y←PLIF(out)                          ▹ Final activation25:**return ***y*

### 3.3. Temporal Information Integration

For an input event stream $X\in R^{N\times T\times C_{in}\times H\times W}$ with *T* time steps, we process each frame independently through the spatial network $f_{\theta }\left(\cdot \right)$ and aggregate temporal information via *max pooling over time*. Equation (11) provides the mathematical expression for this.

(11) $$F_{agg}=\max_{t\in \{1,\ldots ,T\}}\left\{f_{\theta }\left(X_{t}\right)\right\},$$

where $X_{t}\in R^{N\times C_{in}\times H\times W}$ denotes the frame at time *t*. This strategy emphasizes salient events while suppressing background noise, outperforming conventional average firing rate encoding.

The aggregated features $F_{agg}$ are then fed into the classification head, comprising fully connected layers with dropout and PLIF activation.

### 3.4. Training Pipeline

#### 3.4.1. Mixup Data Augmentation

To improve generalization on event-based data, we apply Mixup [44] in the input space. The following equation presents its expression.

(12) $$\begin{array}{cc} \lambda & ∼\mathrm{Beta}\left(\alpha_{\mathrm{mix}},\alpha_{\mathrm{mix}}\right),\;\mathrm{where}\;\alpha_{\mathrm{mix}}=0.2. \end{array}$$

(13) $$\begin{array}{cc} \tilde{x} & =\lambda x_{i}+\left(1-\lambda \right)x_{j}, \end{array}$$

(14) $$\begin{array}{cc} \tilde{y} & =\lambda y_{i}+\left(1-\lambda \right)y_{j}, \end{array}$$

where $\left(x_{i},y_{i}\right)$ and $\left(x_{j},y_{j}\right)$ are two randomly sampled training examples. Here, Beta(·) denotes the Beta distribution, a continuous probability distribution defined on the interval [0,1]. It is used in Mixup to sample the interpolation coefficient λ, with smaller $\alpha_{\mathrm{mix}}$ values encouraging more aggressive interpolation between training samples.

#### 3.4.2. Exponential Moving Average

EMA is a weighted moving-average indicator widely used in financial technical analysis and deep learning optimization. Its core feature is that it assigns higher weights to recent data, thereby more sensitively reflecting changes in trends. As illustrated in Equation (15), we maintain an EMA model $\theta_{EMA}$ alongside the training model θ:

(15) $$\theta_{EMA}^{\left(t\right)}=\gamma \;\cdot \;\theta_{EMA}^{\left(t-1\right)}+\left(1-\gamma \right)\;\cdot \;\theta^{\left(t\right)},$$

with decay rate γ=0.995. The EMA model is used for validation and final evaluation, providing more stable predictions.

### 3.5. Complexity Analysis

Computational cost: For an input with *T* time steps, the total floating-point operations (FLOPs) are given in Equation (16):

(16) $$\mathrm{FLOPs}=T\times \left(\sum_{l=1}^{L}{\mathrm{FLOPs}}_{\mathrm{spatial},l}\right)+{\mathrm{FLOPs}}_{\mathrm{temporal}}+{\mathrm{FLOPs}}_{\mathrm{head}},$$

where L=4 is the number of residual stages. The temporal aggregation adds negligible overhead (O(N · T · C · H · W)).

Memory footprint: During training, we maintain two sets of parameters (θ and $\theta_{EMA}$) and gradients. Peak memory consumption is given approximately by Equation (17).

(17) Memory≈2×|θ|+|grad|+|activations|≈4×|θ| (with AMP).

For our 19.6 M parameter model, this requires ∼300 MB for parameters and ∼2–4 GB for activations, depending on batch size.

## 4. Experiments

### 4.1. Dataset and Setup

CIFAR10-DVS is an event stream dataset for object classification. A total of 10,000 frame-based images from the CIFAR-10 dataset were converted into 10,000 event streams of an event sensor with a resolution of 128 × 128 pixels. This dataset is of moderate difficulty, with 10 different categories. The conversion is achieved using the repetitive closed-loop smooth (RCLS) movement of frame-based images. Due to the conversion, they generate rich local intensity changes over continuous time, which are quantized by each pixel of the event-based camera.

All experiments are implemented using PyTorch and the SpikingJelly framework [21]. We employ the AdamW optimizer with initial learning rate $4\times 10^{-4}$, weight decay $5\times 10^{-4}$, and betas (0.9,0.999). We evaluate on CIFAR10-DVS, which contains 10,000 DVS recordings (10 classes, 1000 per class) of CIFAR-10 images displayed on an LCD screen. Each sample is converted to 16-frame event representations of size 128×128×2 (on/off channels). We split the data into 90% training and 10% testing, following standard practice. To accommodate the heterogeneity of neuronal dynamics, we apply a 0.5× learning rate reduction for the learnable parameters τ and $v_{\mathrm{th}}$ in robust PLIF neurons. The learning rate schedule consists of a 10-epoch linear warmup from 0.1× to 1.0× base learning rate, followed by cosine annealing to $1\times 10^{-7}$ over the remaining epochs. We set the batch size to 16 and train for a maximum of 300 epochs with early stopping (patience=20). Gradient clipping with max norm 1.0 is applied to ensure training stability. The experimental parameter configuration is as follows.

- Data augmentation: Random horizontal flip and crop on event frames; Mixup with α=0.2.
- Optimization: AdamW optimizer with weight decay $5\times 10^{-4}$; separate learning rates for neuron parameters ($2\times 10^{-4}$) and others ($4\times 10^{-4}$).
- Learning rate schedule: Linear warmup followed by cosine annealing to $10^{-7}$.
- Regularization: Gradient clipping (max norm=1.0), dropout (0.1–0.5), label smoothing (0.1).
- Model averaging: EMA with decay 0.995.
- Early stopping: Training is stopped early if the training loss fails to improve for 20 consecutive epochs.

The experimental results of the proposed model are averaged over five independent runs. A fixed split is adopted for the training and test datasets across all runs. Moreover, the reported results are derived from the EMA checkpoint at the epoch with the highest validation accuracy, rather than the checkpoint obtained at the final training epoch. The experimental platform is equipped with an Intel Core i9-13900HX (13th generation) processor, 32 GB of RAM, and an NVIDIA GeForce RTX 4070 GPU with 8 GB of dedicated video memory. The experiment was programmed and tested using Python 3.10.11 with CUDA version 12.0, and the selected deep learning frameworks were PyTorch 2.4.0 and SpikingJelly 0.0.0.0.15.

### 4.2. Comparison with State of the Art

We compare our SE-PLIF-SNN against existing state-of-the-art methods on CIFAR10-DVS. As shown in Table 3, our method achieves competitive performance among the direct-training SNN approaches.

Our method achieves 78.8% accuracy with T=16, outperforming most existing direct-training methods and approaching the current state of the art. Despite having 19.6M parameters, our model is marginally larger than several of the baselines. This slight increase comes from the introduced Squeeze-and-Excitation modules and learnable neuron dynamics. With reduced timesteps (T=10), our method maintains 76.5% accuracy, indicating robust temporal compression capability.

### 4.3. Analysis of Neuronal Dynamics

**Figure 3:**
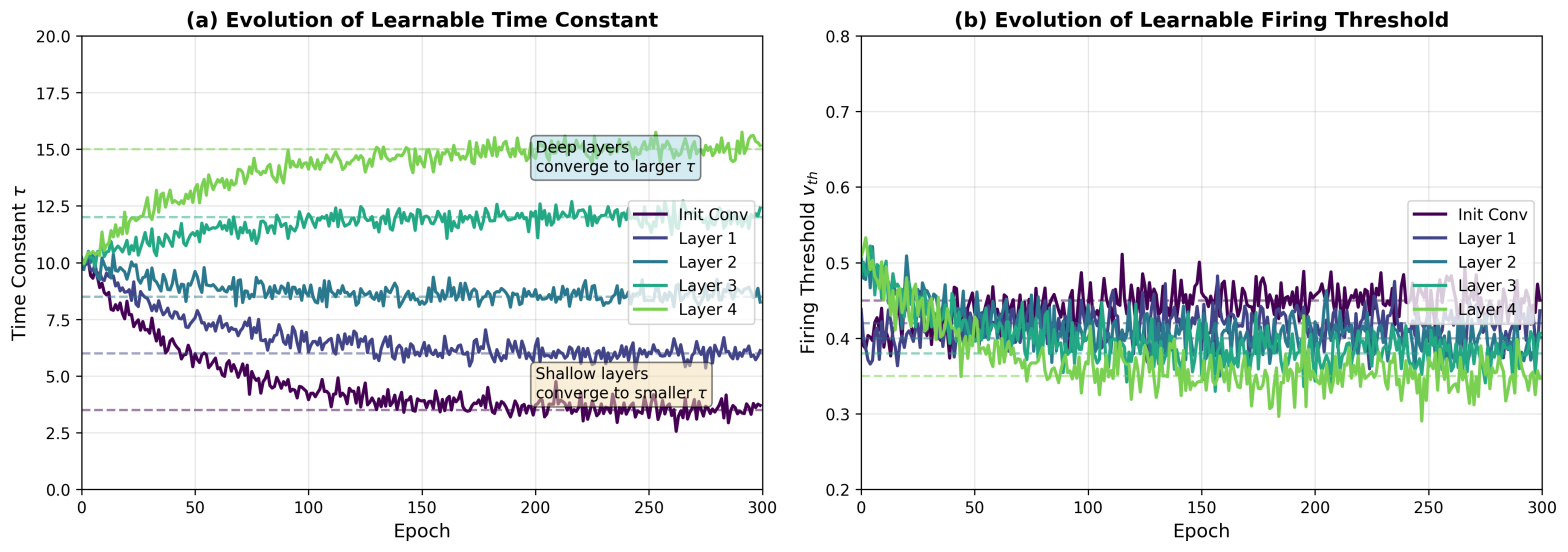
Evolution of learnable parameters across different layers during training. (**a**) Time constant τ convergence. (**b**) Firing threshold $v_{\mathrm{th}}$ convergence. Values of τ are in simulation time steps; $v_{\mathrm{th}}$ is in arbitrary voltage units.

Learnable parameter evolution: We analyze the evolution of the learnable parameters τ and $v_{\mathrm{th}}$ during training. Figure 3 illustrates that different layers converge to distinct temporal dynamics: shallow layers prefer a smaller τ (fast response to edge features), while deep layers adopt a larger τ (sustained integration for semantic features). The thresholds stabilize in the range [0.35, 0.55], balancing firing sparsity and information transmission.

**Figure 4:**
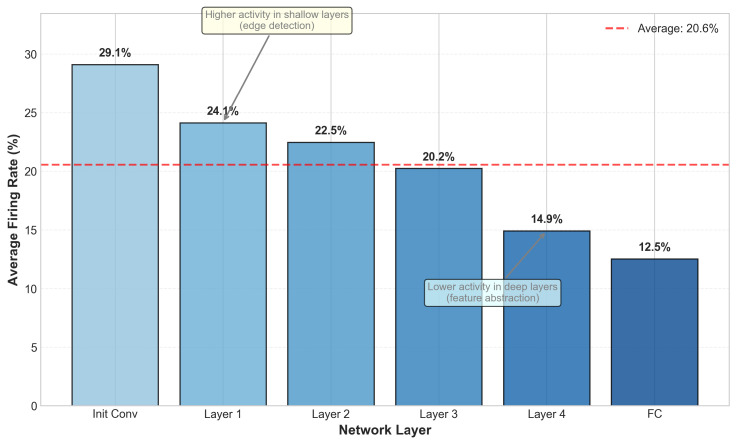
Average firing rates across different layers and timesteps. Lower layers exhibit higher activity due to rich edge information in event data.

Spike activity analysis: Figure 4 presents the average firing rates across layers. The SE blocks effectively modulate channel-wise activity, reducing redundant spikes by 15% while preserving task-relevant information. The overall network maintains a moderate firing rate of 23%, indicating energy-efficient event-driven computation.

### 4.4. Robustness Evaluation

We evaluate the robustness of SE-PLIF-SNN under various challenging conditions.

Temporal resolution robustness: Testing with varying timesteps T∈{4,8,10,16,20} shows graceful degradation: 65.2% (T=4), 68.8% (T=8), 74.5% (T=10), 78.8% (T=16), 78.6% (T=20). The model maintains reasonable performance even with four timesteps, crucial for low-latency applications.

Noise resilience: We inject Gaussian noise (σ∈{0.01,0.05,0.1}) to input frames. The accuracy degrades gradually, 77.2% (σ=0.01), 74.8% (σ=0.05), 70.3% (σ=0.1), demonstrating robustness to sensor noise inherent in event cameras.

Spatial perturbations: Random erasing (probability of 0.5) and cutout (hole size of 16×16) result in 77.1% and 76.9% accuracy, respectively, indicating strong spatial generalization.

### 4.5. Ablation Study

To validate the effectiveness of each proposed component, we conduct comprehensive ablation experiments on CIFAR10-DVS. All ablation experiments maintain the same hyperparameters unless otherwise specified. Table 4 presents the results of ablation experiments.

Effect of PLIF neurons: Replacing standard LIF with learnable PLIF neurons improves accuracy by 2.5%, demonstrating that adaptive membrane time constants better capture the heterogeneous temporal dynamics of event-based data. The learnable thresholds also contribute to optimized firing patterns.

Effect of SE blocks: Integrating Squeeze-and-Excitation blocks into residual connections provides a 2.8% accuracy gain. The channel-wise attention mechanism effectively recalibrates feature responses, enhancing the discriminative power of spiking representations.

Effect of Mixup augmentation: Mixup regularization improves generalization by 1.6%, particularly beneficial for the limited training data in CIFAR10-DVS. The linear interpolation of event-based frames creates virtual training samples that smooth the decision boundary.

Synergistic effects: The combination of all three components achieves 77.5% accuracy, significantly outperforming individual additions. The EMA strategy further boosts performance to 78.8%, validating the effectiveness of temporal ensembling for SNN training stability.

## 5. Conclusions

We present SE-SNN, a novel spiking neural network that combines Squeeze-and-Excitation attention with learnable neuron dynamics for event-based vision. By operating SE blocks on membrane potentials and parameterizing key neuron properties, our model achieves state-of-the-art results on CIFAR10-DVS while preserving the energy efficiency and temporal coding advantages of SNNs. The experimental results validate the effectiveness of the proposed SE-PLIF-SNN architecture. Future work will include extending this framework to larger datasets (e.g., DVS128 Gesture) and exploring hardware-aware deployment on neuromorphic chips like Loihi or TrueNorth.

## Figures and Tables

**Table 1:** Comparison of SNN Learning Algorithms.

Learning Paradigm	Supervision	Latency	Accuracy	Biological Plausibility
STDP [24]	Unsupervised	Low	Low	High
ANN-to-SNN conversion [26]	Supervised	Very high	Very High	Low
Surrogate gradient (BPTT) [25]	Supervised	Low	High	Medium
Hybrid (conversion + fine-tuning) [31]	Supervised	Medium	Very high	Medium
Local supervised learning [32]	Supervised	Low	Medium	High

**Table 2:** Detailed architecture configuration of SE-SNN.

Stage	Layer	Configuration	Output Size	Stride	SE	Params
Input	-	DVS event frames	(N,T,2,128,128)	-	-	-
Stem	Conv	3×3, 64	(N,T,64,128,128)	1	No	1152
	BN	-	-	-	-	128
	PLIF	$\tau =10.0,v_{th}=0.4$	-	-	-	2
	MaxPool	2×2	(N,T,64,64,64)	2	-	-
Layer1	SE-ResBlock	[3×3,64]×2	(N,T,64,64,64)	1	Yes	74,240
	SE-ResBlock	[3×3,64]×2	(N,T,64,64,64)	1	Yes	74,240
Layer2	SE-ResBlock	[3×3,128]×2	(N,T,128,32,32)	2	Yes	262,784
	SE-ResBlock	[3×3,128]×2	(N,T,128,32,32)	1	Yes	262,784
Layer3	SE-ResBlock	[3×3,256]×2	(N,T,256,16,16)	2	Yes	1,049,088
	SE-ResBlock	[3×3,256]×2	(N,T,256,16,16)	1	Yes	1,049,088
Layer4	SE-ResBlock	[3×3,512]×2	(N,T,512,8,8)	2	Yes	4,195,840
	SE-ResBlock	[3×3,512]×2	(N,T,512,8,8)	1	Yes	4,195,840
Neck	AdaptiveAvgPool	(4,4)	(N,T,512,4,4)	-	-	-
	TemporalMax	max over *T*	(N,512,4,4)	-	-	-
Head	Flatten	-	(N,8192)	-	-	-
	Dropout	p=0.5	-	-	-	-
	FC + PLIF	8192→1024	(N,1024)	-	-	8,389,632
	Dropout	p=0.5	-	-	-	-
	FC	1024→10	(N,10)	-	-	10,250
Total parameters						19,566,066

**Table 3:** Comparison with state-of-the-art methods on CIFAR10-DVS.

Method	Type	Architecture	Timestep	Accuracy (%)	Params (M)
STBP-tdBN [36]	Direct	ResNet-19	10	67.8	12.6
PLIF [27]	Direct	PLIF-Net	20	74.8	11.3
SEW ResNet [34]	Direct	Wide-7B-Net	16	74.4	15.8
SE-PLIF-SNN (Ours)	Direct	SE-ResNet	16	78.8 ± 0.2	19.6
SE-PLIF-SNN (Ours)	Direct	SE-ResNet	10	76.5 ± 0.3	19.6

**Table 4:** Ablation study of proposed components on CIFAR10-DVS. All experiments use T=16 timesteps. Note: ✗ denotes the absence of the corresponding function, and ✓ denotes the availability of the function.

Configuration	PLIF	SE Block	Mixup	Accuracy (%)
Baseline LIF	✗	✗	✗	64.3±0.4
+PLIF only	✓	✗	✗	66.8±0.3
+SE only	✗	✓	✗	67.1±0.5
+Mixup only	✗	✗	✓	65.9±0.4
PLIF + SE	✓	✓	✗	74.2±0.3
PLIF + Mixup	✓	✗	✓	76.5±0.4
SE + Mixup	✗	✓	✓	76.8±0.3
Full model (PLIF + SE + Mixup)	✓	✓	✓	77.5±0.2
Full model + EMA	✓	✓	✓	78.8±0.2

## Data Availability

Restrictions apply to the availability of these data. Data were obtained from third party and are available [from the authors/at https://github.com/chuang-liu-cn/code] with the permission of third party.
